# Intervening in the cycle of poverty, poor housing and poor health: the role of housing providers in enhancing tenants’ mental wellbeing

**DOI:** 10.1007/s10901-021-09852-x

**Published:** 2021-06-03

**Authors:** Lisa Garnham, Steve Rolfe, Isobel Anderson, Pete Seaman, Jon Godwin, Cam Donaldson

**Affiliations:** 1 Glasgow Centre for Population Health, Glasgow Centre for Population Health, Olympia Building, Bridgeton Cross, Glasgow, G40 2QH UK; 2 Faculty of Social Sciences, University of Stirling, Stirling, FK9 4LA UK; 3 School of Health and Life Sciences, Glasgow Caledonian University, Cowcaddens Road, Glasgow, G4 0BA UK; 4 Yunus Chair in Social Business and Health and Pro Vice-Chancellor Research, Glasgow Caledonian University, Glasgow, G4 0BA UK

**Keywords:** Health inequalities, Housing, Place, Poverty, Realist, Tenant, Wellbeing, Qualitative

## Abstract

Poverty, poor housing and poor health are complexly interconnected in a cycle that has proven resistant to intervention by housing providers or policy makers. Research often focuses on the impacts of the physical housing defects, particularly upon rates of (physical) illness and disease. There has been comparatively little research into the ways in which housing services can underpin the generation of positive health and, especially, wellbeing. Drawing on qualitative data from 75 tenants in the social and private rented sectors, this paper describes the findings of a research project that tracked tenants’ experiences across their first year in a new tenancy in Greater Glasgow, Scotland. The project collected data on tenants’ perceptions of housing and housing service quality, financial coping and health and wellbeing, which was analysed using the principles of Realist Evaluation to elucidate impacts and causal pathways. Being able to establish a sense of home was key to tenants’ wellbeing. The home provided many tenants with a recuperative space in which to shelter from daily stressors and was a source of autonomy and social status. A sense of home was underpinned by aspects of the housing service, property quality and affordability which are potentially amenable to intervention by housing providers. These findings raise questions about the extent to which social housing providers and the private rental market in the UK are able to meet the needs of vulnerable tenants. They suggest that approaches to housing provision that go beyond providing a basic dwelling are needed to successfully intervene in the cycle of poverty, poor housing and poor health.

## Introduction

Housing is a key social determinant of health (Dahlgren & Whitehead, 1991; Marmot, 2010; WHO, 2008, 2018). It is well known that there are a wide range of ways in which a lack of good quality housing has negative impacts on health and wellbeing (Braubach et al., 2011; Thomson et al., 2013) and that those households with the fewest resources are most likely to be subjected to housing of inferior quality (WHO Europe, 2009). This problem forms part of a cycle of poverty, poor housing and poor health, in which these three features are heavily interwoven (Baker et al., 2014; Bentley et al., 2019; Marsh et al., 2000), and which has proven resistant to effective intervention (Braubach, 2011; Marmot, 2010). The complexity generated by intersecting housing and health inequalities means that the evidence base on the causal pathways between housing and health outcomes is far from complete, particularly in terms of the theoretical connections that might underpin the links between housing and health (Thomson & Thomas, 2015).

Existing research has tended to focus on the impacts of physical housing quality, predominantly on physical health (Bonnefoy et al., 2003; Braubach et al., 2011). Less well researched are the impacts of the less tangible aspects of housing, particularly on mental health and wellbeing (Clapham et al., 2018). This is despite the fact that the pathways from housing to health are as diverse as the experience of housing itself, taking in aspects of comfort, identity and security, as well as the wider neighbourhood and the affordability of the home (Shaw, 2004). Examining these aspects and their impacts on health and wellbeing requires a focus on the ways in which people experience their home, against the background of their individual needs, expectations and capacity. It is these less tangible aspects of the housing experience, and their impacts on mental wellbeing, that this paper sets out to examine, using longitudinal, qualitative data.

In this paper, we approach the concept of health in line with the World Health Organisation’s definition: “a state of complete physical, mental and social wellbeing, and not merely the absence of infirmity” (WHO, 1946). We use the term wellbeing to mean a combination of positive psychological state and a functional balance between individual resources and challenges (Dodge et al., 2012). Although subjective wellbeing does have some limitations, it is an important short-term indicator of the impacts of day-to-day life on health outcomes across the lifecourse, and there is a growing body of evidence demonstrating the correlation between wellbeing and with other measures of health (Oguz et al., 2013; Steptoe et al., 2015), including physical health outcomes (Topp et al., 2015).

There is a clear role for housing in underpinning inhabitants’ wellbeing, particularly through the generation of ‘ontological security’ (Giddens, 1984, 1991); that is, “the feeling of well-being that arises from a sense of constancy in one’s social and material environment which, in turn, provides a secure platform for identity development and self-actualization” (Padgett, 2007, p.1926). In order to fulfil this role, housing must provide constancy, a place in which day-to-day routines can be formed, a place in which inhabitants can exercise control and a place that is free from surveillance. In so doing, it supplies a secure base from which people can develop self-confidence and social identity, and, in turn, enhances wellbeing (Dupuis & Thorns, 1998; Padgett, 2007; Saunders, 1989).

The concept of the ‘psycho-social benefits of home’ further develops this framework for understanding the pathways from the ontological security provided by housing to inhabitants’ health and wellbeing. Key to these pathways are experiences of autonomy within the home, the home as a haven and the home as a source of social status (Hiscock et al., 2001; Kearns et al., 2000, 2012). Despite this potential, vulnerable tenants often struggle to establish a sense of home in low income housing, particularly in the less secure Private Rented Sector (PRS) (Soaita & McKee, 2019; Woodhall-Melnik et al., 2017).

Whilst longitudinal analyses of the impacts of physical housing improvements provide a useful starting point in developing theoretical pathways between housing and health (Thomson & Thomas, 2015; Willand et al., 2015), there is still a need to more fully explore causality in relation to the less tangible aspects of housing and home, particularly in their impacts on wellbeing. Moreover, in order for housing research to be of practical use to housing policy makers and practitioners, these pathways need to be extended ‘upstream’ to include the ways in which the organisational policies and practices of housing providers feed through into low income tenants’ housing experiences and, in turn, shape their day-to-day wellbeing.

Deepening our understanding of some of the longer and more complex causal pathways between housing, poverty and health is particularly important given the contemporary shift in housing markets across the global North. Over the past decade, the Global Financial Crisis (GFC) has restricted home ownership options (Pittini et al., 2017) and reinforced the growth of the PRS. Across much of Europe, this has taken place in a context of declining funding for and residualisation of the Social Rented Sector (SRS), restricting access to good quality, affordable housing for low income households (Poggio & Whitehead, 2017).

Against this background, in the UK (as elsewhere) many social housing providers have been driven towards more market-based strategies (Gruis, 2005; Morrison, 2016), whilst a number of mission-driven organisations have emerged in the PRS, seeking to support the growing number of low income households in the sector (De Decker, 2012; Shelter Scotland, 2015). This has resulted in new approaches to housing provision and support for low income households, with implications for wellbeing, health and inequality which are, as yet, largely unknown. Whilst these broader developments may reinforce cycles of poverty, poor housing and poor health, they also offer fresh intervention opportunities to counter these intersecting inequalities. However, this requires research that draws out the most effective and meaningful methods of intervention to improve health and wellbeing through housing provision.

This paper explores the impacts of approaches to rented housing provision on low income tenants’ wellbeing, by drawing on the longitudinal, qualitative data gathered as part of the mixed methods ‘Housing through Social Enterprise’ study (Garnham & Rolfe, 2019), which followed a cohort of new tenants in social and private rented accommodation across the first year of their tenancy. Although this study took place in Glasgow, Scotland, the aim is to elucidate some of the general causal pathways through which housing provision impacts on the wellbeing of low-income tenants, to improve our ability to effectively intervene in the cycle of poverty, poor housing and poor health.

## The Scottish context

**Fig. 1:**
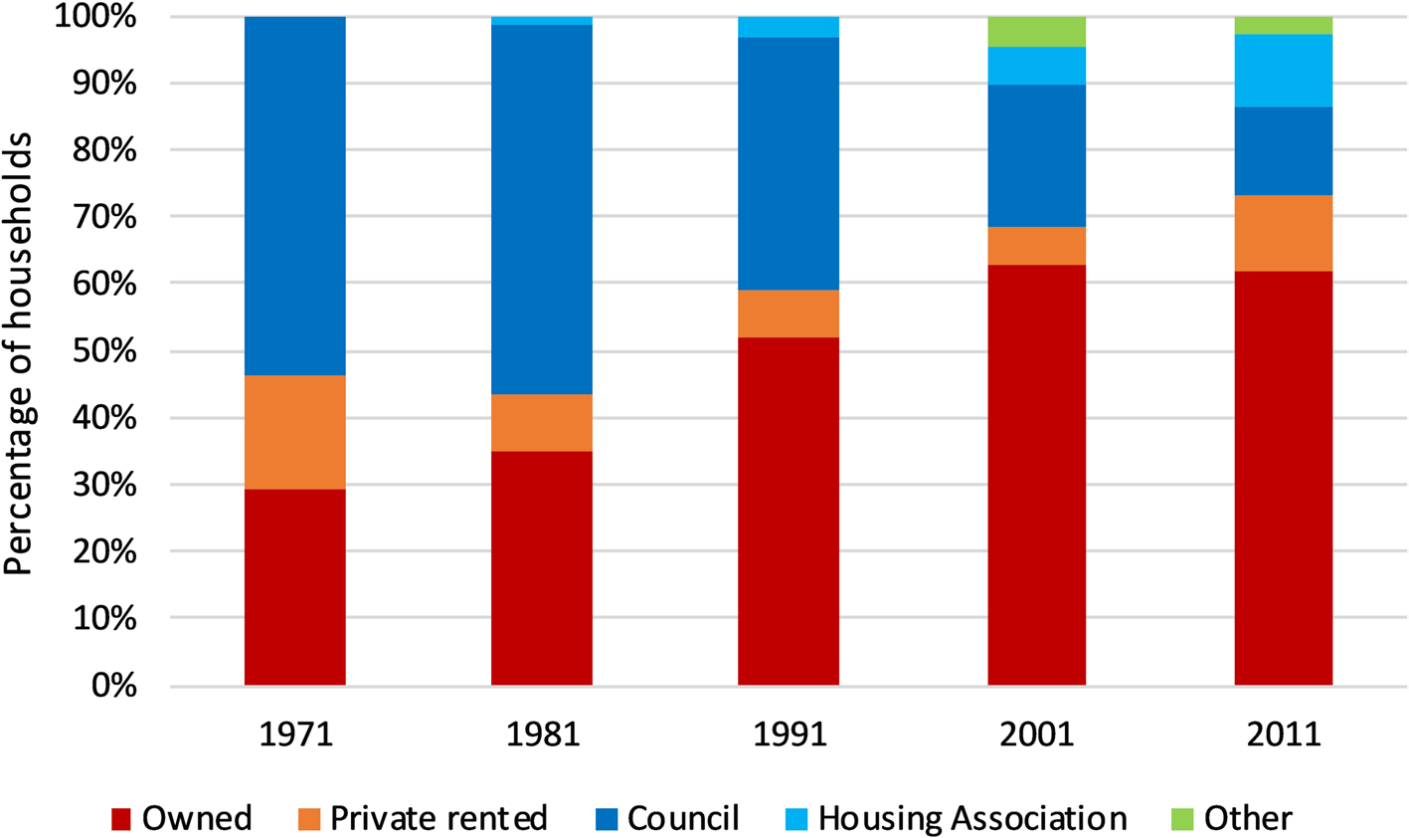
Housing sectors in Scotland, 1971–2011 (*Source*: Census data)

Historically, Scotland’s low income households were served by its large PRS, in which conditions were typically poor. In the post-war period, the vast majority of PRS properties were purchased and either renovated, or demolished and replaced by publicly owned housing, in a slum clearance programme designed to provide affordable, quality homes for all. Erosion of this housing sector began in the 1980s, as a policy change enabled tenants to purchase their homes from local government. Since the 1990s, local government has also transferred stock to not-for-profit ‘social’ landlords (typically Housing Associations) and some, such as Glasgow City, no longer own any housing (Fig. 1).

These sectoral shifts have generated a recognition that there is an undersupply of affordable housing in Scotland, especially in the SRS, and particularly in the context of changes to the UK’s benefits system (Dunning et al., 2020; Powell et al., 2015). There is, therefore, an increasing proportion of low income households being diverted from an underfunded SRS to the PRS, across a range of age groups and household types (Bailey, 2018). Given their socio-economic position and increased risk of experiencing poor health, these households are particularly vulnerable to the potentially damaging impacts of poor quality housing, both in the SRS and the PRS.

Unlike SRS landlords, which have always had to be registered *and* adhere to detailed operational parameters, it is only since 2013 that PRS landlords have needed to be registered in Scotland. Further, tenancy agreements in the PRS are significantly less secure than in the SRS and this remains the case despite the introduction of the new ‘private residential tenancy’ in Scotland in late 2017. While SRS tenancies are effectively permanent as long as rent payments are maintained, PRS landlords have the right to evict tenants at relatively short notice and for no cause. Moreover, while rents set by SRS landlords are ‘capped’ close to the local rates set for Housing Benefit, there are no such restrictions on PRS rents, which are market driven. Finally, while SRS landlords, by their very nature, specifically cater for low income households, PRS landlords and their agents routinely exclude those on low or unstable incomes (Hoolachan et al., 2017).

Despite these disadvantages, the PRS still has something to offer low income households in Scotland, beyond mere availability and speed of acquisition, including greater choice in the size, style and location of property, as well as a greater degree of flexibility (Rolfe et al., 2019). While, generally speaking, the SRS offers tenants greater potential to personalise the interior of a property to their taste, in many cases the PRS offers properties with higher quality interior finishes from the outset (Garnham & Rolfe, 2019). That being said, there is significant variability in housing and service quality across both of these sectors, particularly for low income households.

Against this background, a number of innovative models of housing provision and tenancy support have emerged in the PRS in Scotland in recent years, which seek to better meet the needs of low income households forced into the PRS. This context offers significant opportunities to explore the experiences of low-income households, in both new and more traditional models of housing provision, and the impacts that various models and styles of housing provision might have on the housing experience and, in turn, on tenant wellbeing.

## Methods

The Housing through Social Enterprise study followed 75 tenants through the first year of their tenancy in Greater Glasgow, Scotland between September 2016 and June 2018. Participants were recruited through three housing organisations, which operate across the SRS and PRS. All three are not-for-profit and have social missions that prioritise low-income and vulnerable households, as outlined in Box 1. Together they provided an opportunity to explore a range of practices and impacts in working with such households.

Ethical approval was provided by the [University Ethics Committee]. New tenants were given information about the study by their housing organisation before being contacted by telephone by the researchers to explain the study and ascertain whether or not they wanted to participate. Participants were interviewed at three time points over the first year of their tenancy, as shown in Table 1, and informed consent was obtained at each Wave.

**Box 1:** Outline of participant housing organisations

**Housing Association** Community-based Housing Association, providing social rented housing in one neighbourhood. Also operates subsidiary regeneration organisation focused on employment and community development. This organisation aims to provide affordable housing to low-income households, and contribute to community sustainability and regeneration through non-housing activities. Owns and manages around 5500 properties **Letting Agency** Social enterprise letting agency which manages property for traditional PRS landlords, and purchases and refurbishes property to rent to low-income households. Has social mission to provide high quality housing in the PRS to vulnerable households and provides intensive tenancy support service, partly funded from service charge income. Manages around 250 properties and owns a further 200 **Rent Deposit Schemes** Voluntary sector organisation running two Rent Deposit Schemes (RDS), facilitating access to the PRS for households at risk of homelessness. Provides deposit guarantee to landlords on behalf of prospective tenants, to enable vulnerable households without savings to access tenancies. Tenants pay deposit in instalments over the first year of their tenancy, rather than paying it up-front, and are provided with tenancy support. Combined, the schemes support around 100 people into new tenancies each year

**Table 1:** Data collection

Wave	Time point	Method	Focus	N
1	Start of new tenancy	Quantitative telephone interview	Previous housing situation and baseline health and wellbeing	121
2	2–4 months into new tenancy	Quantitative and qualitative face-to-face interview	New housing situation and short-term impacts on health and wellbeing	75
3	9–12 months into new tenancy	Quantitative and qualitative face-to-face interview	Established housing situation and long-term impacts on health and wellbeing	45

The qualitative interviews at waves 2 and 3 were semi-structured, covering aspects of the housing service, community and social networks, financial circumstances and health and wellbeing. We used follow-up questions to explore tenants’ perceptions of the impacts that various aspects of the housing experience were having on their mood, their quality of life and how they felt about themselves. We specifically asked participants whether they felt their health and wellbeing had changed through the course of their tenancy and what they attributed that change to.

Whilst the study was mixed methods, this paper focuses on our analysis of the qualitative data gathered at Waves 2 and 3; the quantitative analysis has been presented elsewhere (Rolfe et al., 2020). Our quantitative analysis identified correlations between aspects of housing and health and wellbeing, providing initial, tentative causal pathways. Note that, whilst wellbeing is clearly influenced by low income, our quantitative analysis specifically identified that these correlations existed when controlling for income. The qualitative analysis presented here seeks to build on and refine our understanding of these causal pathways between housing and wellbeing by exploring the particular mechanisms at play and the contextual factors that influence their operation (Clapham et al, 2018; Pawson & Tilley, 1997).

Our analysis utilised the principles of Realist Evaluation to examine ‘what works, for whom, in what circumstances’ (Pawson, 2013; Pawson & Tilley, 1997). Using Nvivo, interview transcripts were coded to identify combinations of contexts, interventions, mechanisms, and health and wellbeing outcomes in linked dyads or triads (Jackson & Kolla, 2012). There are significant challenges in distinguishing between these different aspects of causal pathways, particularly separating interventions from underlying mechanisms and identifying relevant contextual factors (Dalkin et al., 2015). To overcome these issues, this paper follows Punton et al. (2016) in presenting the interventions separately. Thus, the analysis attempts to identify the contextual factors in terms of tenant characteristics and resources, which combine with interventions by the housing provider, to trigger mechanisms which generate health and wellbeing outcomes.

Whilst this research also identified aspects of the wider neighbourhood in which the home is situated that impacted on many tenants’ ability to feel at home (Rolfe & Garnham, 2020), this paper focuses on those elements of housing provision which are more amenable to intervention by housing organisations. The mechanisms are primarily conceptualised using the notions of home and ontological security, although the analysis also attempts to refine these ideas as a ‘middle-range theory’ (Merton, 1968) of housing as a social determinant of health.

## Results

**Fig. 2:**
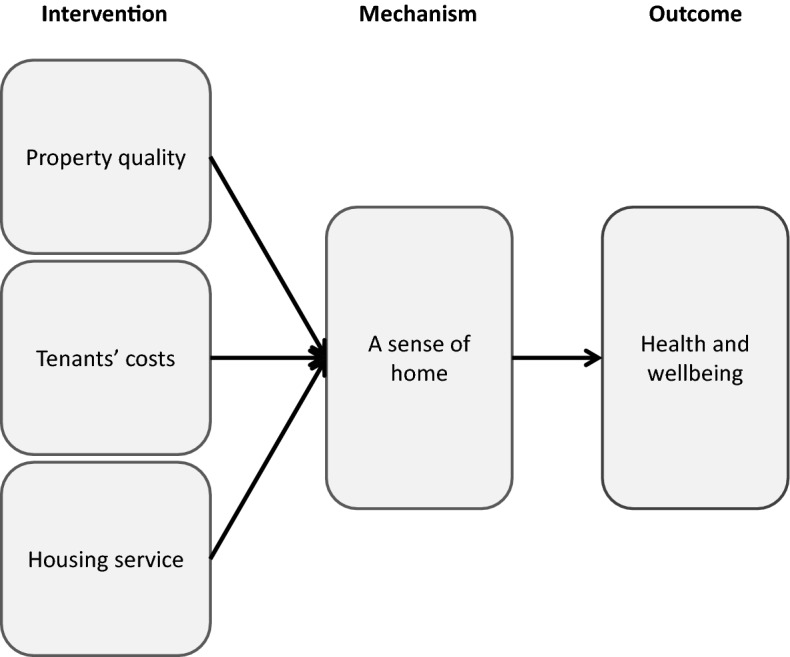
Findings summary

The key mechanism through which housing impacted on the wellbeing of participants was by supporting or inhibiting their ability to establish a sense of home in their new tenancy. This, in turn, was underpinned by three aspects of their housing: property quality; tenancy costs; and the housing service, as summarised in Fig. 2. The remainder of this paper describes the ways in which each of these aspects affected tenants’ sense of home, before outlining the mechanisms through which a sense of home contributed to tenants’ wellbeing.

### Property quality

**Fig. 3:**
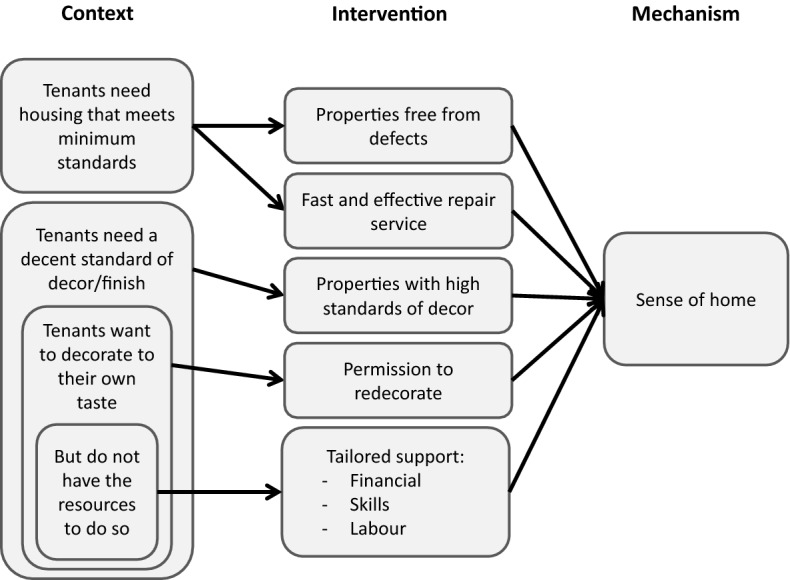
Property quality

Tenants’ ability to establish a sense of home was influenced by two aspects of property quality. The first related to property defects, where basic standards were not met. These properties had serious, ongoing repair and maintenance issues, requiring regular contact with the housing provider, repeated visits from tradespeople and disruption to daily life. The second related to what might be considered to be ‘cosmetic’ aspects of the property – plasterwork, flooring, décor and furniture – which were nevertheless pivotal to many participants’ experiences in their new tenancy. Figure 3 summarises the main pathways from these aspects of property quality, through the actions of housing organisations, to an established sense of home for tenants.

Although almost 80% of participants rated the physical quality of their property as good or very good, a significant number nevertheless described an array of physical defects with their homes during their qualitative interviews. These included dampness from water ingress or poor ventilation, vermin or other kinds of infestation, poor electrical installations, and poor soundproofing and energy inefficiencies, including draughty windows and doors, absent or poor insulation and old, ineffective or poorly controlled heating systems. Added to these defects with the building fabric were issues with essential fixtures and fittings, including taps, showers, floor boards, light fittings, and so on.

Where property quality was good at move-in and the maintenance service was fast and effective, repairs were rapid, infrequent and caused little disruption to tenants. However, where the property had been allowed to fall into disrepair or the maintenance service was unresponsive, slow or ineffective, property quality became a significant problem. This was especially the case when the maintenance needed was extensive, affecting many rooms and leaving few areas to which the tenant could retreat, or where the same repair was attempted several times, leaving the tenant with little faith that it would ever be complete and their privacy would be regained.

Not only did these experiences leave tenants in sub-standard properties for long periods, they also prevented them from making changes to establish a sense of home, as this participant describes:

I'm dis-attached […] there's no connection between me and this place. […] It's nothing to be proud of, I have done nothing here. Because I started to gather, I bought a few tools, I brought here ladder and a few other things I borrowed from friends but there was no point to do anything because they agreed to replace the floorboards. And I thought, okay, so there is no point to doing anything else in the flat because I need to wait till they finish.

Tenants’ concerns about property quality often extended beyond the basic fabric of the building, to include general decorative order and cleanliness. An urgent need for cleaning or decoration at move-in was not only expensive and time-consuming, but also demoralising and stressful at a time of upheaval and instability. Renewed paintwork, wallpaper, flooring and even plastering were sometimes required to bring properties up to a reasonable standard, before tenants could furnish, unpack and settle in their new home.

While these aspects of a property may be considered ‘cosmetic’, it is clear from this research that failing to meet a basic minimum standard of décor has a significant impact on tenants’ ability to establish a sense of home. This is especially the case where tenants do not have the financial means, skills and expertise, physical capability, or support networks needed to carry out this work themselves:

The walls in here are pretty bad and at one point I phoned the housing officer and I says to her, listen, I'm going to have to give you that house back. That’s far too much work for me. ‘Cause I thought she said to me she would plaster the walls and then when I came in, they weren’t plastered. So it had big holes and all that’s still to be done up the top […] I can’t, I’ve nobody to help me or nothing and I'll, there’s nothing I can do to that house.

Where housing organisations provided tenants with properties of a high standard of finish, or forewarned tenants about the property condition and supported them in finishing the property once moved in, most were able to settle quickly and establish a sense of home.

In addition to a minimum standard of decor, the majority of tenants also needed to be able to “put their own stamp on the place” for their new tenancy to feel like home. For many this simply meant hanging pictures and furnishing the property to suit their needs, for others it extended to painting a couple of rooms, and others still were keen to undertake more extensive work. However much involvement they wanted in redecorating, this process was most successful when housing providers supported tenants in doing so.

I'm limited to what I can do. […] I would have to ask their permission first [to decorate]. I wouldn’t want to do something like that because, knowing my luck, [...] you go and do the work and then once all the works have been done, [the landlord] wants the property back. Whereas, if I had my own, if I had permanent accommodation and did the work then, yes, it feels like home.

For some, like the participant above, all that was required was a secure, long-term tenancy and the permission to redecorate, while others required financial support in purchasing materials and someone with the necessary skills to help them.

In summary, all tenants required a property that met minimum quality standards and offered a basic standard of décor. Many also wanted to personalise the property to feel settled and some needed support from their housing provider to do this. The form of this support depended greatly on tenants’ expectations and previous housing experiences, as well as their capacity and support network of friends, family or agencies. Importantly, these early experiences of the property often shaped tenants’ approach to the tenancy throughout the first year, substantially influencing their ability to settle and feel at home in the longer term.

### Tenancy cost

**Fig. 4:**
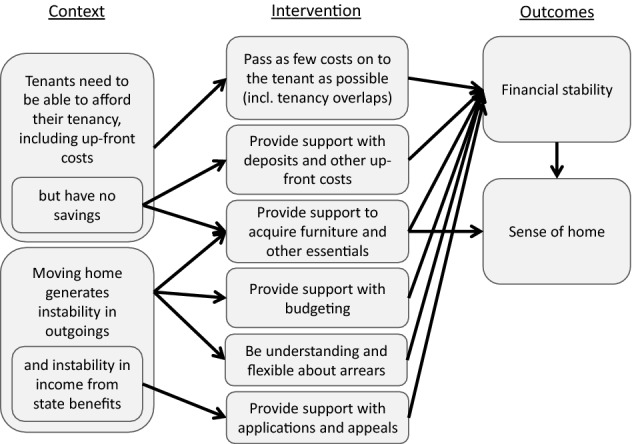
Tenancy cost

Almost all participants in this research were on a low or very low income: 79% of participating households lived on less than 50% of the Scottish median household income after housing costs. Finances therefore had a significant impact on their quality of life, health and wellbeing, including their ability to settle into their new property and establish a sense of home. As outlined in Fig. 4, there were two key ways in which a new tenancy contributed to these impacts: the costs of moving and establishing a home; and the uncertainty and instability of outgoings (and, in many cases, income) brought about by the process of moving. Together, these contributed to tenants’ financial stability which, in turn, had a significant impact on their ability to establish a sense of home in their new tenancy.

The primary cost typically associated with a new tenancy is rent. However, the overwhelming majority of participants in this study reported no problems with paying their rent. This was partly because just over half received Housing Benefit, which mostly or fully subsidised their rent and was typically paid from government directly to the landlord. It was also partly a function of tenants prioritising rent above other expenditure, including heating and food.

Rather, a wide range of other costs associated with a new property were the main cause of financial concerns for participants in this study, particularly where large sums were required up front. This included landlords’ requests for a deposit and first month’s rent in advance, the need to pre-load gas and electricity meters (particularly in winter), the need for furniture (often essential items like mattresses and white goods) and the cost of basic decorating (including covering bare floorboards and putting up curtains/blinds). A number of participants described having to spend long periods without these essential aspects of home or extend themselves deeply into debt to secure them:

I think the last time you came I had no furniture, no curtain, no carpet and basically I was just trying to cope. But now I think the house is well suited for living for anybody. […] Then I was struggling just to get my apartment furnished but now I am in debt, but the pressure of accumulated debt is making it worse, coupled with the fact that my income dropped.

These costs were exacerbated where a participant’s previous tenancy overlapped with their new one, for example if work was still being done in the new property by the landlord after the tenancy began, which prevented them from moving in for a period of time. For many tenants in this study, these costs added to everyday financial strains caused by low incomes, coupled with the costs of travel, food, supporting children and other family and friends.

Tenants settled more easily and started to feel at home where housing organisations minimised these initial costs or supported tenants in meeting them. This included ensuring the property was adequately decorated and furnished and that repair work was finished before the lease began. This allowed the tenant to move into a ‘ready-to-go' property, avoiding significant outlays in overlapping rent and bills, or investment in essential ‘finishing’ items for the property. A number of tenants were also supported to pay their deposit in instalments, either as an addition to their rent payments, or through a ‘bond’ arrangement with a third-party Rent Deposit Scheme. Alongside landlords’ acceptance of rent paid in arrears (often driven by the time taken for Housing Benefit applications to be processed) these arrangements allowed tenants on very low incomes to successfully access and establish their new tenancy.

For other tenants, the unpredictability and instability of outgoings and incomes during this period caused significant anxiety. Problems included uncertainty around costs in a new property (e.g. utilities, furniture needed, travel costs to work) and therefore being unable to budget effectively for costs over the first few months. This was particularly problematic for people moving into a new area or from outside the UK, and for those moving household as well as property (e.g. moving out of a family home, moving in with a partner). Only once the financial instability of moving settled down were participants able to start to feel at home:

I'm on top of everything, as I say I've paid all my bills now, so it's like you can just sit back and relax now, you know what I mean, it's your home, your house, you do what you want. Before I didn't have that, so it's a major difference, I feel it's a major difference.

The depth and duration of financial instability was often exacerbated for those in receipt of welfare benefits, which have to be reassessed upon moving home. This typically involved lengthy delays in benefit payments, leading most study participants to accumulate at least some debt.

Much of the support that housing organisations provided in relation to financial instability involved helping with benefit applications and appealing benefit rejections, as well as supporting tenants to anticipate costs and budget for them, including meeting debt liabilities. However, the single biggest contribution that housing organisations could make to tenants’ financial stability was by not becoming a source of instability themselves. Practices such as preventing an overlap in tenancies, or fully explaining how heating and hot water systems worked, worked well to prevent tenants from accumulating unexpected rent and utilities bills.

Where debts had begun to accumulate, intensive debt collection practices often reinforced tenants’ sense of instability and precarity. The approach to rent arrears, in particular, had a significant impact on tenants’ ability to establish a sense of home. While the vast majority of participants in this study stated they had no difficulties paying their rent, a substantial number nevertheless accumulated rent arrears. This was typically caused by an overlap in tenancies (often only a few days) and the fact that Housing Benefit can usually only cover one tenancy at any time. This created periods of rent liability which tenants had limited resources to pay and which they were often unaware of until much later. Where these arrears were treated sensitively, with patience, and where the cause was properly explained to tenants, the impact was often minimal:

I don’t feel as pressurised into, when I'm speaking about my £80 in rent arrears, you know, other [housing providers] would be a lot quicker off the mark. But they’re understanding at this time of year [Christmas] and they're happy enough. And they know with my issues, my depression and my anxiety, even some days I don’t go out the house, I can't even go to the shop some days it's that bad. So they are aware and they do help with that.

Where the causes or even the scale of the arrears were not properly explained, or more aggressive recovery processes were used, this proved very disruptive, as this participant describes:

I’d already had arrears and then waiting the six weeks [for a new Housing Benefit application], they transferred my case to court, which I was really angry about. Because they could see that I was waiting on the Housing Benefit, they knew it was coming, they just hadn’t got it yet. […] I’ve always paid ten pound a week all for my arrears, and [my housing officer] says, ‘well, I just need to [refer it to court]. Protocol.’ […] I was really, really stressed and upset. Just at the thought of going to court and stuff like that, she wasn’t explaining much to me, she just kept sending out these big horrible letters that were just putting the fear of God into me […] I just felt like I explained everything and they were just making it worse for me, when I was trying to make it better.

Where the housing provider was unsupportive, problems with financial instability typically persisted many months into the tenancy and impacted heavily on tenants’ ability to establish a sense of home over the long term.

### Housing service

**Fig. 5:**
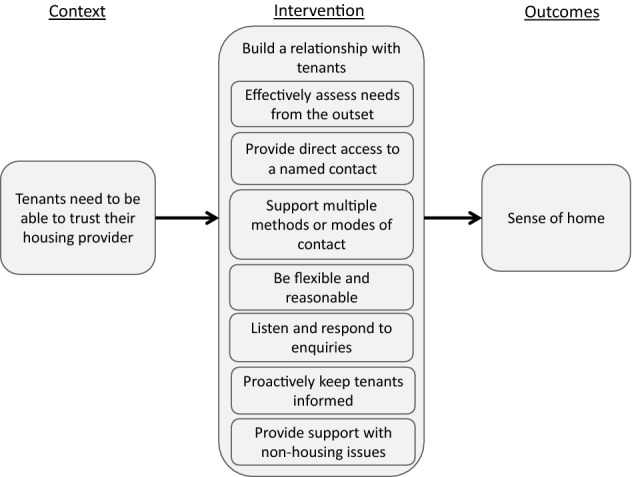
Housing service

Tenants needed to be able to trust their housing provider in order to establish a sense of home, and a number of service aspects contributed to or undermined that trust. Fundamentally, housing providers needed to commit to building a relationship with their tenants, primarily through offering a ‘person-centred’ service that focussed on meeting tenants’ needs, as outlined in Fig. 5.

There were two core facets of this relationship from tenants’ perspectives. The first was the experience of interacting with their key contact – their first port of call for information or support and the person who would contact them from the housing organisation. The second was the experience of interacting with the wider organisation, including other members of staff. The majority of participants described their relationship with their key contact as central to their experiences of housing service:

The difference between my housing officer and my girlfriend's housing officer is night and day. One doesn't care or pretends she doesn't care, whereas mine does care, she does got that wee bit, you know. I asked her, at the beginning, to give me details of the house and all that kinda thing, no problem, she phoned me back when she said she would. Whereas the other housing officer is just the total opposite, it's like she doesn't care.

The ways in which tenants were able to keep in touch with their key contact was of primary importance in building this relationship. Tenants especially valued having a named contact, who they had met in person at or before the start of their tenancy, who had got to know them and who they could contact directly. Having a variety of modes of contact was important; being restricted to one or two methods that did not suit the tenant (e.g. in person by appointment only, or by web form with replies by email) was often problematic.

Having made contact, tenants needed their concerns or requests to be answered promptly and with as much accurate information as possible. Being kept up to date with developments, in a proactive way, was essential. Together, these experiences built respect and trust between the tenant and the key contact, which enabled them to offer the tenant effective support with their tenancy and beyond. Instances in which communication had broken down and damaged this relationship were much more frequently commented upon than instances in which communication was seamless and effective.

Many of the tenants in this study required more extensive support in establishing their tenancy, from completing application forms, to setting up utilities payments, to organising removals. While some had networks to draw on for that support, others only had housing organisation staff to turn to. Where the organisation supported them in establishing their tenancy, for example by helping with paperwork, liaising with third parties or giving advice and general support, this provided invaluable assistance to tenants in stabilising their tenancy and also served to build trust.

For many tenants, this relationship strengthened throughout the first year of the tenancy, particularly where housing organisation staff were able to offer appropriate support on non-housing issues. This included diverse activities, from helping with transport to hospital, to supplying household basics like bedding, to being someone “on the end of the phone” when a tenant needed support:

I mean, [my key contact] kinda spoke me through a few things. I think she maybe thought that I was taking the break up [from my partner] a wee bit harder than maybe somebody of my age should be taking it. But, you know, I'm a softie, so there you are. But no, they were there when I needed them and I don't need them so much now.

Tenants who were supported in this way particularly valued being offered support without having to explicitly ask, which could only happen because of the person-centred approach of their key contact. However, it is important to note that this sensitive, tailored approach also involved not being too intrusive:

It doesn’t feel like a home where they are every two months, checking on you. I think, it makes you think that, oh, that’s not your home, that’s our property and we come to check on you, like checking on your personal life, how you live your life, and how you decorate your own house and your own flat. Yeah, every time they were coming I had to remove all my decoration from the wall.

Flexible, supportive relationships with key contacts needed to be reflected in organisational procedures and practices, including maintenance procedures, arrears management, application processes and tenancy agreements. Organisational procedures that were overly onerous, formal or rigid tended to restrict the ability of key contacts to offer meaningful help, exerted pressure on tenants and created distance between tenants and the housing organisation. This tenant describes the negative impact of organisational procedures around tenancy agreements on their sense of home:

I thought maybe because they know that I’d be here like long term then they’d draw up another tenancy, but they still just go on the month to month. So, there’s that and all. And they only need to give me a month’s notice as well. [So it’s just] somewhere to stay, like living in somebody else’s house.

Overall, where tenants’ relationships with their housing organisation were positive, they described staff as friendly, personable, respectful, reassuring and approachable, which in turn reflected on how they felt about their home. Where these experiences were negative, they talked of an organisation that was too formal, uncaring, inflexible, unfriendly or selfish, which inhibited tenants’ ability to feel at home.

### Impacts on wellbeing

**Fig. 6:**
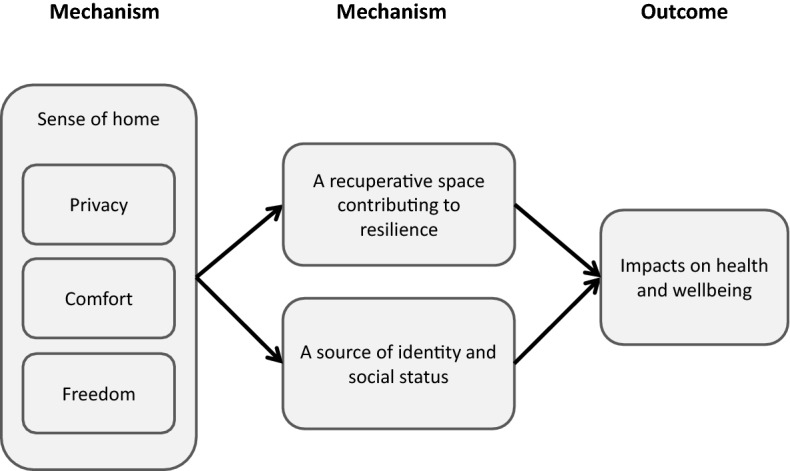
Sense of home

When describing what having a sense of home felt like, participants talked about one of three things. Firstly, a sense of privacy, protection and peace, perhaps best encapsulated by the description of the home as a “sanctuary” or, in line with Kearns et al. (2012), a “haven”. Secondly, the comfort that the home could provide, often being described as cosy and warm (both literally and figuratively). Thirdly, and stemming partly from the first two, the ways in which the home could offer a sense of freedom. Where the property provided a comfortable sanctuary, participants felt they could do whatever they wanted within their home, and it acted as a base from which they could tackle any problems they were facing and to which they could retreat when they needed to recuperate. There were therefore two distinct pathways through which tenants’ sense of home impacted on their mental wellbeing, summarised in Fig. 6. The first was the potential for the home to contribute towards their resilience, by providing a recuperative space in which they could shelter and recover from day-to-day stressors. The second was the potential for the home to contribute towards their self-esteem, pride and identity.

In terms of a recuperative space, participants described their home as a place in which they could relax and de-stress, being able to “come in, shut the door and be yourself” in privacy. In this way, the home became protective of their mental wellbeing. For many, this provided both a physical space and a state of mind in which they could look to the future, offering stability and a sense of direction:

Well, one of the things that makes it feel like home is that I’m at liberty to do whatever I want to do without anybody checking me or questioning me. Secondly I’m at liberty to sleep as much as I want to sleep and if I’m hungry I can easily walk into my kitchen, make food for myself in a serene environment. […] I’ve acquired a home and this is where I will settle down and get married and have my children until when I’m ready to move to a bigger apartment, so that gives me a happiness to plan. So the quality of life is there, because what gives you the happiness is when you have something to plan and make your future plan, so having that here has given me stability.

Moreover, for those who were proud of their home, either in appearance or function, it contributed to their self-worth. This was particularly the case for those who had put significant resources into improving the property. The sense of achievement this provided was a source of wellbeing that participants were surrounded by and reminded of on a day-to-day basis:

Because I was in prison, I didn’t get the chance to have privacy. So see within this house, shutting that door, I get all the privacy I want. And I feel as if, because I’ve done it all up, I feel as if I’ve accomplished something. I'm proud of what I’ve done. My family are all dead proud of me. And this place is letting me get my head back together again, ‘cause my head was wasted. Wasted. I was away in jail for years and years and years. And this place has really kept me together.

For others, however, the property was not a home, but an active source of stress and negative feelings. Where the property was uncomfortable or did not feel private, for example, participants became increasingly unhappy, anxious and worried:

Two months without a cooker, countless weeks where the boiler would break down and I wouldn't have hot water and I wouldn't have any gas for cooking […] so aye, that was really kinda detrimental to health. I lost a bit of weight through it as well and my mental health suffered as well, ‘cause it was as if they didn't care. So I was going to work depressed, coming back depressed, looking at the depressing house.

These kinds of circumstances prevented the property from serving as a recuperative space and impacted negatively on participants’ self-worth, leaving many feeling “in limbo” and unable to plan. This often had additional impacts on social relationships, in that tenants could not host guests and felt they were imposing on friends/family while escaping their own property:

I'm not maybe depressed but I'm ashamed of the flat, I can't invite people here, my social life is just limping, you know. It's not something, it's fun when you may do something around you just to create the space you live in, and I'm just now in suspension again, so I just can't find another word for that.

This, in turn, further threatened their sources of support and resilience in dealing with life’s challenges.

A sense of home, then, was the central mechanism through which participants’ housing situation impacted upon their wellbeing. Establishing a sense of home could have a direct, positive impact on mental wellbeing *and* provide a source of resilience against outside threats to wellbeing. Further, it was particularly important that tenants were able to establish a sense of home quickly, as once negative experiences in the property began to take hold, it was often difficult for a tenant to recover and establish a sense of home later on in the tenancy.

## Discussion

This research has illuminated a number of key pathways through which tenants’ broader experiences of housing and housing service impact on mental wellbeing. These findings highlight the fact that much of the impact of housing, at least when it is of a tolerable standard, is on mental health and wellbeing and that many of these causal pathways are substantially shaped by the broader support offered by housing providers. This provides a significant extension of the concept of housing as a social determinant of health, moving beyond much of the existing evidence base regarding the effects of physical housing defects on illness and disease (Braubach et al., 2011; WHO Europe, 2007), to demonstrate the role of the less tangible aspects of housing experience in impacting positively and negatively on mental wellbeing (Bond et al., 2012).

In particular, these findings elaborate the role of ontological security and a sense of home as pathways linking aspects of housing experience to tenants’ wellbeing, through the pathways shown in Fig. 6. Of particular note is that fact that participants in this study emphasised the importance of home, not just as a secure base from which identity and a positive sense of self can be built and maintained, but as a space in which the self can be defended from outside stressors. For low income households, these stressors are likely to be multiple. Thus, the ability of tenants to settle in a new tenancy and feel at home is important not just as an end in itself, but a means of enhancing the wellbeing of low those living in low income households, via the ‘psychosocial benefits of home’ (Hiscock et al., 2001; Kearns et al., 2000, 2012). Utilising a Realist lens, these findings develop the middle range theory (Merton, 1968; Pawson & Tilley, 1997) of a sense of home as a mechanism which can generate improved mental wellbeing, applicable across a range of settings.

The causal models outlined in this paper elucidate some of the ways in which housing organisations can intervene to enable low income tenants to develop a sense of home, alongside the contextual factors which influence their effectiveness and require consideration in targeting resources. In this respect, the study both supports and moves beyond Kearns et al.’s (2000) contention that factors detracting from psychosocial benefits may be more important than those that contribute to them. Combinations of tenant characteristics, such as low income and lack of social support, together with problematic housing experiences, such as poor move-in condition and property-centred services, can undermine tenants’ sense of home, creating negative psychosocial effects and damaging health and wellbeing. However, these findings show that there is also a clear potential for housing organisations to intervene positively in such situations to support tenants and facilitate positive psychosocial effects. We have mapped out the pathways through which housing organisations can support tenants to establish a home in which there is constancy, day-to-day routines can be formed, they can exercise control and that is free from surveillance, thus enabling the development of ontological security and enhancing wellbeing (Dupuis & Thorns, 1998; Padgett, 2007; Saunders, 1989).

Nevertheless, this analysis has a number of limitations. Firstly, sthis paper only provides direct evidence of the impacts of housing on mental wellbeing amongst a relatively small cohort of tenants in the west of Scotland. Care is therefore required in extrapolating these findings to other households in other localities. However, our application of a Realist analytical framework has enabled the development of models with potential for broader applicability. The middle range theory centred around the role of ‘home’ as a mechanism that shapes tenant wellbeing offers sufficient abstraction to be relevant to other housing contexts. Alongside this, the elaboration of contextual factors and interventions which trigger this mechanism provide a specificity which would benefit from further examination and refinement.

Secondly, this research focused on housing organisations which espouse a social mission, aiming in different ways to support vulnerable households to access and sustain housing. In this respect, the study provides more limited evidence of the impact of the less tangible aspects of the housing experience on tenants’ sense of home and wellbeing in more typical PRS tenancies, which tend to be driven by profit rather than a social mission. Further research in this area would be of value, particularly given the growth in the numbers of low-income households in the PRS who do not have the support of organisations with a social mission.

## Conclusion

These findings demonstrate the potential for successful intervention in the cycle of poverty, poor housing and poor health. This paper has outlined ways in which low-income and vulnerable households are at risk of negative impacts on wellbeing from housing problems, reinforcing the evidence that low-income households are likely to experience particular difficulties in creating a ‘home’, given poor property conditions and insecurity of tenure (Soaita & McKee, 2019). Moroever, our research has highlighted ways in which such risks might be mitigated, across both private and social rented sectors, and perhaps most importantly, the ways in which housing can underpin the development of positive wellbeing.

These findings present a challenge to housing organisations as to how they might shape their services to build positive relationships with tenants, address housing costs beyond rent levels and provide properties in a condition that is ‘liveable’, not just ‘lettable’. There are particular challenges around how such an approach might be resourced in socially-orientated housing organisations and what incentives (if any) there may be to encourage for-profit housing organisations to work in this way.

In the context of ongoing changes to the housing market in the UK (Bailey, 2018) and across Europe (Pittini et al., 2017; Poggio & Whitehead, 2017), these findings raise questions as to whether it is possible to re-orientate the priorities and practices of for-profit landlords towards the needs of low income households, as seems to be the Scottish Government’s current approach (Scottish Government, 2013). Moreover, whilst the SRS in Scotland is starting to expand once again, the long-term effects of decades of decline and residualisation has left the sector primarily housing those in greatest need, creating resource challenges in delivering person-centred housing services.

Hence, whilst this study provides useful evidence regarding the significant potential for housing organisations to intervene positively to disrupt the cycle of poverty, poor housing and poor health, it raises questions about the extent to which housing providers in either rental sector are equipped to undertake this work at present. It may therefore take concerted and coordinated effort amongst policy-makers, housing organisations and public health practitioners to build on this understanding of the health and wellbeing implications of a sense of home, in order to tackle entrenched housing and health inequalities.

## Data Availability

The data underlying this study has not yet been archived in a repository. Parties interested in accessing the data should contact the corresponding author.

## References

[CR1] Bailey, N. (2018). The divisions within ‘Generation Rent’: poverty and the re-growth of private renting in the UK. (Paper presented at *Social Policy Association Conference 2018.* York)

[CR2] Baker E, Mason K, Bentley R, Mallett S (2014). Exploring the bi-directional relationship between health and housing in Australia. Urban Policy and Research.

[CR3] Bentley R, Baker E, Aitken Z (2019). The ‘double precarity’ of employment insecurity and unaffordable housing and its impact on mental health. Social Science & Medicine.

[CR4] Bond L, Kearns A, Mason P, Tannahill C, Egan M, Whitely E (2012). Exploring the relationships between housing, neighbourhoods and mental wellbeing for residents of deprived areas. BMC Public Health.

[CR5] Bonnefoy X, Braubach M, Krapavickaite D, Ormand D, Zurlyte I (2003). Housing conditions and self-reported health status: A study in panel block buildings in three cities of Eastern Europe. Journal of Housing and The Built Environment.

[CR6] Braubach M (2011). Key challenges of housing and health from WHO perspective. International Journal of Public Health.

[CR7] Braubach M, Jacobs DE, Ormandy D (2011). Environmental burden of disease associated with inadequate housing.

[CR8] Clapham D, Foye C, Christian J (2018). The concept of ssubjective well-being in housing research. Housing, Theory and Society.

[CR9] Dahlgren G, Whitehead M (1991). Policies and strategies to promote social equity in health: Background document to WHO – Strategy paper for Europe.

[CR10] Dalkin SM, Greenhalgh J, Jones D, Cunningham B, Lhussier M (2015). What’s in a mechanism? Development of a key concept in realist evaluation. Implementation Science.

[CR11] De Decker P (2012). Social Rental Agencies: An Innovative Housing-led Response to Homelessness.

[CR12] Dodge R, Daly A, Huyton J, Sanders L (2012). The challenge of defining wellbeing. The International Journal of Wellbeing.

[CR13] Dunning, R., Ferrari, E., Hoolachan, J., Keskin, B., Moore, T., O’Brien, P., Powell, R. (2020). Affordable Housing Need in Scotland Post-2021. *Shelter Scotland*.

[CR14] Dupuis A, Thorns D (1998). Home, home ownership and the search for ontological security. The Sociological Review.

[CR100] Garnham, L., & Rolfe, S. (2019). Housing as a social determinant of health: evidence from the housing through social enterprise study. *Glasgow: Glasgow Centre for Population Health*.

[CR15] Giddens A (1984). The constitution of society.

[CR16] Giddens A (1991). Modernity and Self Identity: self and society in the late modern age.

[CR17] Gruis V (2005). Financial and social returns in housing asset management: Theory and Dutch housing associations' practice. Urban Studies.

[CR18] Hiscock R, Kearns A, MacIntyre S, Ellaway A (2001). Ontological security and Psycho-Social benefits from the home: Qualitative evidence on issues of tenure. Housing, Theory and Society.

[CR19] Hoolachan J, McKee K, Moore T, Soaita AM (2017). ‘Generation rent’ and the ability to ‘settle down’: economic and geographical variation in young people’s housing transitions. Journal of Youth Studies.

[CR20] Jackson SF, Kolla G (2012). A new realistic evaluation analysis method: Linked coding of context, mechanism, and outcome relationships. American Journal of Evaluation.

[CR21] Kearns A, Hiscock R, Ellaway A, Macintyre S (2000). 'Beyond Four Walls'. The Psycho-social benefits of home: Evidence from West Central Scotland. Housing Studies.

[CR22] Kearns A, Whitley E, Bond L, Tannahill C (2012). The residential psychosocial environment and mental wellbeing in deprived areas. International Journal of Housing Policy.

[CR23] Marmot M (2010). Fair society: Health lives. Strategic review of health inequalities in England post-2010.

[CR24] Marsh A, Gordon D, Heslop P, Pantazis C (2000). Housing deprivation and health: A longitudinal analysis. Housing Studies.

[CR25] Merton R (1968). Social theory and social structure.

[CR26] Morrison N (2016). Institutional logics and organisational hybridity: English housing associations’ diversification into the private rented sector. Housing Studies.

[CR27] Oguz S, Merad S, Snape D (2013). Measuring National Well-being - what matters most to personal well-being?.

[CR28] Padgett D (2007). There’s no place like (a) home: Ontological security among persons with serious mental illness in the United States. Social Science & Medicine.

[CR29] Pawson R (2013). The science of evaluation: A realist manifesto.

[CR30] Pawson R, Tilley N (1997). Realistic evaluation.

[CR31] Pittini A, Koessl G, Dijol J, Lakatos E, Ghekiere L (2017). The state of housing in the EU 2017.

[CR32] Poggio T, Whitehead C (2017). Social housing in Europe: Legacies, new trends and the crisis. Critical Housing Analysis.

[CR33] Powell, R., Dunning, R., Ferrari, E. & McKee, K. (2015). Affordable housing need in scotland: Final report—September 2015. *Shelter Scotland*.

[CR103] Rolfe, S. & Garnham, L. (2020). Neighbourhood impacts on wellbeing: Investigating the role of housing among low income tenants. *Social Inclusion (special issue),**8*(3), 102–112

[CR101] Rolfe, S., Garnham, L., Anderson, I., Seaman, P., Godwin, J. & Donaldson, C. (2019). Hybridity in the housing sector: Examining impacts on social and private rented sector tenants in Scotland. *Housing Studies,**35*(6), 1050–1072 10.1080/02673037.2019.1648770 PMC7279951 32514222

[CR102] Rolfe, S., Garnham, L., Godwin, J., Anderson, I., Seaman, P. & Donaldson, C. (2020). Housing as a social determinant of health and wellbeing: developing an empirically-informed realist theoretical framework. *BMC Public Health*. 20 (1138) 10.1186/s12889-020-09224-0 PMC7370492 32689966

[CR34] Punton M, Vogel I, Lloyd R (2016). Reflections from a Realist Evaluation in Progress: Scaling Ladders and Stitching Theory.

[CR35] Saunders P (1989). The meaning of ‘home’ in contemporary english culture. Housing Studies.

[CR36] Scottish Government (2013). A place to stay, a place to call home: A strategy for the private rented sector in Scotland.

[CR37] Shaw M (2004). Housing and public health. Annual Review of Public Health.

[CR38] Scotland S (2015). Social models of letting agencies.

[CR39] Soaita, A. M. & McKee, K. (2019). Assembling a ‘kind of’ home in the UK private renting sector. *Geoforum**103 *, 148–157

[CR40] Steptoe A, Deaton A, Stone AA (2015). Subjective wellbeing, health, and ageing. Lancet (London, England)..

[CR41] Thomson H, Thomas S (2015). Developing empirically supported theories of change for housing investment and health. Social Science and Medicine.

[CR42] Thomson, H., Thomas, S., Sellstrom, E. & Petticrew, M. (2013). Housing improvements for health and associated socio-economic outcomes. *Cochrane database of systematic reviews (Online),* 2. 10.1002/14651858.CD008657.pub2 PMC12551615 23450585

[CR43] Topp CW, Ostergaard SD, Sondergaard S, Bech P (2015). The WHO-5 well-being index: a systematic review of the literature. Psychotherapy and Psychosomatics.

[CR44] WHO Europe. (2007). *Large analysis and review of European housing and health status (LARES: Preliminary overview*. WHO Europe.

[CR45] WHO Europe. (2009). *Social inequalities and their influence on housing risk factors and health*. WHO Europe.

[CR46] WHO (1946). Constitution of WHO: principles. Geneva: World Health Organization. Available from: https://www.who.int/about/mission/en/.

[CR47] Willand N, Ridley I, Maller C (2015). Towards explaining the health impacts of residential energy efficiency interventions—a realist review. Part 1: Pathways. Social Science and Medicine.

[CR48] Woodhall-Melnik J, Hamilton-Wright S, Daoud N, Matheson FI, Dunn JR, O’Campo P (2017). Establishing stability: exploring the meaning of ‘home’ for women who have experienced intimate partner violence. Journal of Housing and the Built Environment.

[CR49] WHO (2008). Closing the gap in a generation: health equity through action on social determinants of health.

[CR50] WHO (2018). WHO Housing and health guidelines: Recommendations to promote healthy housing for a sustainable and equitable future.

